# Interactions among vascular plants, bryophytes, and lichens in grassland communities along elevational gradients

**DOI:** 10.1007/s00114-025-02049-0

**Published:** 2025-12-02

**Authors:** Michele Mugnai, Luca Di Nuzzo, Andrea Beltramini, Paride Balzani, Clara Frasconi Wendt, Giulio Ferretti, Alice Misuri, Renato Benesperi, Daniele Viciani, Lorenzo Lazzaro

**Affiliations:** 1 Department of Biology, University of Florence, Florence, Italy; 2 BIOME Lab, Department of Biological, Geological and Environmental Sciences, Alma Mater Studiorum-University of Bologna, Bologna, Italy; 3 Faculty of Fisheries and Protection of Waters, South Bohemian Research Center of Aquaculture and Biodiversity of Hydrocenoses, University of South Bohemia in České Budějovice, Zátiší 728/II, 38925, Vodňany, Czech Republic; 4 Botanical Garden “Giardino dei semplici”, University Museum System, University of Florence, Firenze, Italy

**Keywords:** Assembly rules, Biodiversity, Functional diversity, SEM, Vegetation

## Abstract

Grasslands in temperate Europe are key biodiversity hotspots, supporting not only a rich vascular flora but also diverse communities of bryophytes and lichens. However, the processes shaping lichens and bryophytes in these ecosystems, particularly along environmental gradients, remain understudied. We investigated the relative roles of abiotic factors (elevation and rock abundance) and biotic drivers (vascular plant cover and height) in determining both the taxonomic and functional diversity of bryophytes and lichens in two protected grassland sites in central Italy. Across 80 plots spanning wide elevational gradients, we measured species richness and functional diversity for both bryophytes and lichens, and applied piecewise structural equation modeling to test causal relationships. Our results revealed that vascular plant structure (particularly vegetation cover and height) was the main determinant of lichens and bryophytesdiversity. Both facets of diversity in bryophytes and lichens were negatively affected by dense and tall vascular vegetation, likely due to shading and competition for space. Abiotic factors, such as elevation and rock abundance, influenced lichens and bryophytesonly indirectly, by altering vascular plant structure. An exception was bryophyte species richness, which was directly promoted by rock abundance through increased habitat heterogeneity and reduced vascular plant competition. Site-specific effects, likely related to substrate type (calcareous vs. siliceous), also played a significant role, particularly in shaping bryophyte communities. These findings suggest that biotic interactions, especially competition with vascular plants, play a more central role than direct abiotic filtering in determining lichens and bryophytesdiversity in grassland ecosystems. Our study provides a functional, multi-taxa framework for understanding elevational patterns of lichens and bryophytesdiversity and highlights the importance of considering vegetation structure in grassland conservation and management strategies.

**Supplementary Information:**

The online version contains supplementary material available at 10.1007/s00114-025-02049-0.

## Introduction

Natural and semi-natural grasslands are habitats characterized by high diversity and are considered as biodiversity hotspots in temperate Europe (Wilson et al. 2012; Biurrun et al. 2021). One of the causes of this high biodiversity is linked to the multitude of microhabitats present in grassland ecosystems, justifying their high conservation value (Habel et al. 2013; Dengler et al. 2014).

Alongside the richness of vascular plant species, grassland habitats are also important reservoirs for the biodiversity of the non-vascular vegetation, in particular lichens and bryophytes (Deane-Coe and Stanton 2017; Martin and Mallik 2017; Löbel et al. 2006; Turtureanu et al. 2014; Biurrun et al. 2021), and invertebrates (Zulka et al. 2014). A multitude of complex processes were depicted between vascular plants and other organisms in grasslands, for example, a direct effect of vegetation structure on ant species and trait composition (e.g., Frenette-Dussault et al. 2013; Mugnai et al. 2021). At the same time, competition and facilitation interactions are known to occur among vascular plants, bryophytes, and lichens in grasslands, albeit they are far less studied (Wietrzyk-Pelka et al. 2021). On the one hand, in grasslands, vascular plants could exert a strong competitive pressure on bryophytes and lichens. Vascular plants produce litter that could eventually cover lichens and bryophytess (Ellis et al. 2011), reduce the available space for their occupancy (Ingerpuu et al. 2005; Boch et al. 2018), and create a shading effect that strongly reduces the amount of light reaching the underlying lichens and bryophytes communities (Jägerbrand et al. 2012; Graglia et al. 2001). Such competitive interactions could have a negative effect on both richness and abundance of bryophytes (Löbel et al. 2006; Müller et al. 2012; Klink et al. 2017) and lichens (Bruun et al. 2006, Cornelissen et al. 2001), and could also play an important role in determining the structure of such communities (Fergus et al. 2017). On the other hand, at least at low vascular plant cover, there is also some evidence of facilitation processes occurring between these organisms, especially between vascular plants and bryophytes (Ingerpuu et al. 2005). For example, the cover of vascular plants could reduce the underlying temperature and wind speed that in turn reduce evaporation, allowing lichens and bryophytes to experience longer periods of hydration (Ingerpuu et al. 2005). The relative importance of competition and facilitation could also vary along environmental gradients (Ingerpuu et al. 2005) or vegetation succession (Hagenberg et al. 2022; García de León et al. 2016; Dettweiler-Robinson et al. 2013).

Species richness is generally considered one of the main measures of biodiversity (i.e., taxonomic facet of diversity), but it may not always depict changes in biodiversity (e.g., Chase et al. 2019). On the contrary, functional traits (i.e., functional facet of diversity) have a universal approach, as they do not rely on species taxonomic identity. They inform on ecosystem multifunctionality and services, and they more easily and rapidly respond to environmental changes (Violle et al. 2014; Gagic et al. 2015; Mugnai et al. 2022). Moreover, both lichens and bryophytes respond strongly to environmental gradients such as climate (Matos et al. 2017) since they functionally differ from vascular plants in their lack of specialized structures to regulate rates of water loss from their tissues (i.e., poikilohydry) and poor ability to take up nutrients from the soil. Additionally, it has been demonstrated how rocks and stones strongly influence lichens and bryophytes’ diversity of grasslands, since they consist of suitable microhabitats for many terricolous and epilithic species (Bergauer et al. 2022; Cole et al. 2008; Hespanhol et al. 2011). Among the functional traits of lichens and bryophytesrelated to environmental changes, bryophytes growth form and shoot length are related to water retention and temperatures (Glime 2017), frequency of sexual reproduction is influenced by humidity and stresses (Austrheim et al. 2005), while leaf length could enhance the functional distinction between species. Regarding lichens, growth form is influenced mainly by water availability and temperatures (Nascimbene and Marini 2015; Di Nuzzo et al. 2021), and type of reproduction is influenced by habitat stability and temperature (Seymour et al. 2005; Hurtado et al. 2019; Di Nuzzo et al. 2021). Despite the demonstrated importance of including functional traits in multi-taxa studies, a limited number of authors have addressed biodiversity following this approach (see Roos et al. 2019; Asplund et al. 2022), and none have focused on grassland ecosystems.

One of the most important natural environmental gradients is the elevational one. Such gradients have proved to be pivotal to exploring the effect of climate-induced changes on biodiversity since they encompass the variation of abiotic factors in a relatively short distance, simulating wider climatic and ecological succession in time (Di Nuzzo et al. 2021). Moreover, the consideration of multiple taxa within the same community may depict more precisely the processes acting on biodiversity and better discern whether some abiotic factors are predictors of diversity (Sundqvist et al. 2013; Peters et al. 2016). Previous studies have measured the response of vascular plant functional diversity to elevational gradients, with contrasting conclusions (see Bello et al. 2013; Pescador et al. 2015) and few studies have considered the functional diversity of lichens and bryophytes (see Asplund et al. 2022), despite their importance as components of many ecosystems, especially at high elevations: for example they have a pivotal role in nutrients cycles and litter decomposition, represent microhabitats or shelters for many microorganism groups, and represent food resources for terrestrial invertebrates (e.g. Asplund and Wardle 2017).

Hence, we aimed at assessing whether elevational patterns of lichens and bryophytes diversity were directly driven by the abiotic factors (e.g., elevation and rock abundance) or mediated by biotic drivers (i.e., characteristics of vascular plant vegetation through competition processes). To disentangle how such processes shape lichens and bryophytes communities, we followed multiple approaches considering both taxonomic (with species diversity limited by the number of available niches due to homogeneous environment or strong biotic interactions) and functional (with trait divergence when competition with vascular plants prevails over environmental filtering) facets of diversity. We hypothesized that in a grassland ecosystem, the predominance of vascular vegetation plays the role of the main predictor of lichens and bryophytes diversity, affecting not only species diversity, as already demonstrated (e.g., Bergauer et al. 2022; Hagenberg et al. 2022), but also their functional traits, thus resulting in a lower importance of abiotic conditions such as elevation compared to competition in shaping these communities.

## Methods

### Study sites

**Fig. 1:**
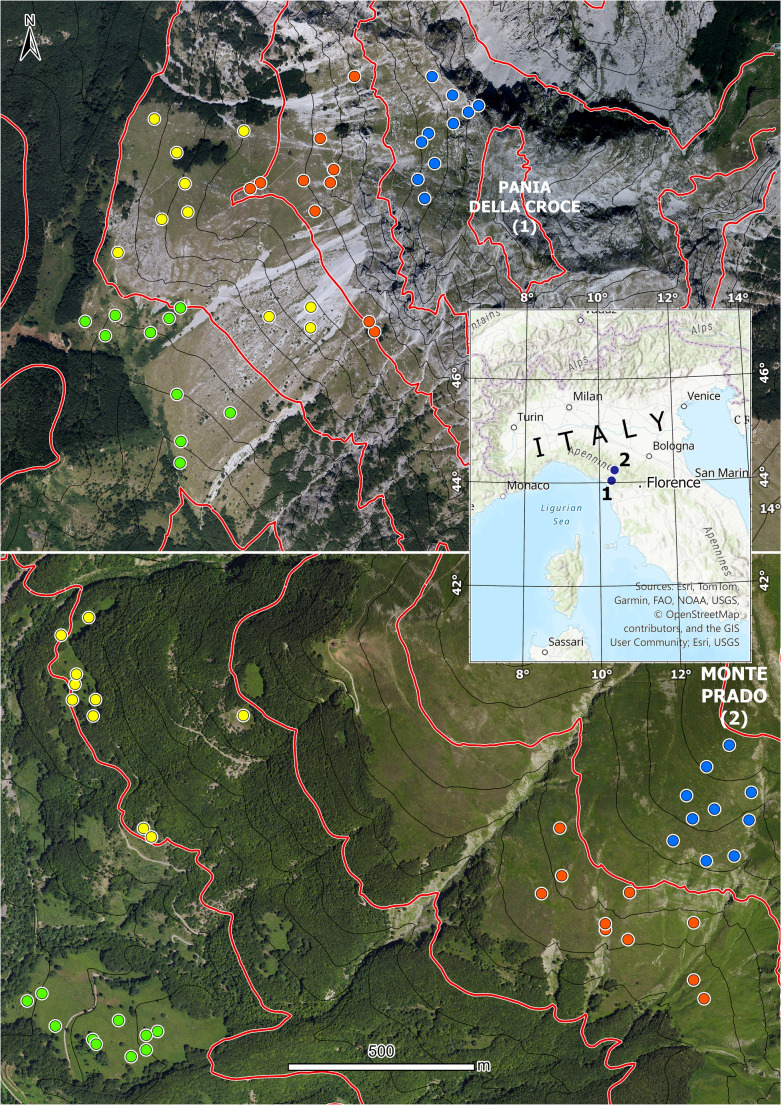
Map of the study sites (1 for Pania della Croce and 2 for Monte Prado) and spatial distribution of sampling points. Elevational belts are delimited by red contour lines and sampling points are color-coded depending on the belt (in order green, yellow, red and blue from the lower to the upper elevational belts)

This study was conducted in two mountains (Fig. 1) included in protected sites situated in central Italy (Tuscany): Pania della Croce (44.035756°N, 10.319390°E) and Monte Prado (44.248877°N, 10.405821°E). These mountains are located within the regional park of Alpi Apuane and within the national park of Appennino Tosco-Emiliano, respectively. Pania della Croce reaches 1858 m a.s.l. with a 5 °C annual mean temperature and 1208 mm of annual precipitation, while Monte Prado’s maximum elevation is 2054 m a.s.l. and is characterized by an annual mean temperature of 3 °C and annual precipitation of 1056 mm (Karger et al. 2017). The two sites differ in the type of substrate: Pania della Croce is mainly constituted by calcareous rocks, while Monte Prado by a siliceous substrate.

Two areas of about 0.7 km^2^ were delimited in both sites, in both cases situated on west-facing slopes of the mountains and covering an elevational gradient of 800 m (from 1000 to 1800 m a.s.l. for Pania della Croce and from 1200 to 2000 m a.s.l. for Monte Prado). Each area included grassland patches classified as semi-natural dry grasslands and scrubland facies on calcareous substrates (Festuco-Brometalia order, important sites for orchids; habitat code 6210*) and siliceous alpine and boreal grasslands (habitat code 6150) according to the HaSCITu (Habitat in the Sites of Conservation Interest in Tuscany) program (http://www.regione.toscana.it/-/la-carta-degli-habitat-nei-siti-natura-2000-toscani). In both areas, grazing and other anthropogenic disturbances (e.g., mowing) are absent, and the treeline reaches around 1120 m a.s.l. for Pania della Croce site and 1690 m a.s.l. for Monte Prado site.

### Sampling

We surveyed in 2020 (in May for Pania della Croce and in June for Monte Prado) 40 2 × 2 m quadrat plots for each area (80 in total) distributed along the elevational gradient. To ensure an even spatial distribution of replicates, each area was divided into four elevational belts, each one covering 200 m of elevation. Within each belt, ten plots were randomly placed (with a minimum distance of 20 m and a maximum distance of 350 m between two plots). In each plot, vegetation and environmental variables were surveyed following the EDGG protocol (European Dry Grassland Group, see Dengler et al. 2016), with vascular plants, epigeic bryophytes, and epigeic lichens identified at species level and their abundances estimated as percentage of coverage down to 0.1%. Community of vascular plants was also characterized by the measurement of structural parameters of vegetation, namely plant cover (total coverage in the plot of vascular plant species in %) and plant height (mean vegetation height measured in five random points). As environmental variables (see Dengler et al. 2016 for methodology), we estimated the fraction of soil covered by rocks (percentage cover of rocks with diameter > 63 mm after virtually removing all vegetation and litter), microrelief (perpendicular distance in cm between lowest and highest ground points), and elevation registered at the plot level with a portable GPS with 4 m of horizontal precision.

We selected a set of functional traits of bryophytes (life form, shoot length, frequency of sporophyte, and maximum leaf length), and lichens (growth form, type of reproduction, type of photobiont, and presence or absence of secondary metabolites), responding well to micro-environmental variation (Giordani et al. 2014; Hurtado et al. 2019; Sulavik et al. 2021; Di Nuzzo et al. 2021). The traits were measured at species level (thus not considering intraspecific trait variation) and in case of numerical traits we used the mean value assuming to be representative for mature healthy individuals. For lichens and bryophytes, we considered all the species occurring in the plots and we characterized them by a set of functional traits retrieved from Hill et al. (2007) for bryophytes and Nimis and Martellos (2020) for lichens. Lichen growth forms were modified from Nimis and Martellos (2020). Species belonging to the genus *Cladonia* with a persistent primary thallus were labeled as “mixed”.

Bryophyte and lichen diversity patterns were evaluated using species richness for the taxonomic facet, as it has been widely considered an adequate index for lichens and bryophytes in these contexts (e.g., Ingerpuu et al. 2005; Bruun et al. 2006; Boch et al. 2016; Boch et al. 2018), and Mean species Pairwise Dissimilarity (hereafter MPD) for the functional one. MDP measures the expected functional dissimilarity between a pair of species randomly selected from the community (Bello et al. 2016). Moreover, functional MPD is unrelated to species richness, allowing the measurement of the functional diversity with an index which is independent of the taxonomic facet (Clarke and Warwick 1998; Bello et al. 2016). MPD was calculated using functional dissimilarity matrix with Gower distance (Gower 1971) and weighing it for abundances of species (Bello et al. 2016). In order to perform statistical analyses and differentiate the functional space of empty or poor-species communities, a value of 0 has been attributed to plots with no species (empty functional space) and a value of 0.0001 to plots with one species (point-like functional space).

### Statistical analyses

For all analyses, we used the statistical software R (version 4.1.2, R Development Core Team, http://www.R-project.org*).*

**Fig. 2:**
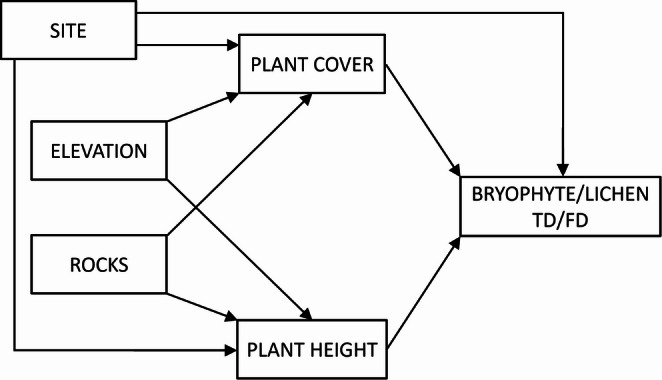
Structure of the a priori model for piecewise structural equation modelling (SEM) exploring the effects of site, elevation, rock abundance, and plant community characteristics on bryophyte and lichen facets. Arrows show hypothesized causal relationships

Since abiotic (i.e., elevation and rocky fraction of soil) and biotic (i.e., vascular plant, bryophyte, and lichen communities) predictor variables were expected to show complex interrelationships, confirmatory path analyses (piecewise structural equation modelling, SEM) were applied in separate models to explore their causal relationships with bryophyte and lichen diversity. An initial model containing the possible causal drivers of lichens and bryophytes communities and their interrelationships was set up based on the current knowledge (Fig. 2). We hypothesized that the abiotic variables (elevation, rock abundance and site) influenced primarily the structure of vascular plant community (vegetation height and cover), which in turn influenced the taxonomic and functional diversity of bryophytes and lichens. Model fit was assessed using Fisher’s C statistic, where *p* > 0.05 indicates that the data are well represented by the model. Piecewise SEMs were based on generalized linear models (GLM) using the R package ‘piecewiseSEM’ (Lefcheck 2016). We fitted linear models for vegetation cover and vegetation height, while for species richness and functional diversity of lichen and bryophytes, we performed a series of generalized linear models with different error family distributions. In particular, we used the Poisson family for lichen richness through the ‘glm’ function of the ***stats*** package and a Negative Binomial (‘nbinom1’) family for bryophyte richness using the ‘glmmTMB’ function from ***glmmTMB*** package (Brooks et al. 2017). Regarding the functional traits of both bryophytes and lichens, we used a Beta family (‘ordbeta’) through the ‘glmmTMB’ function. When using ‘glmmTMB’ models, we set microrelief as random effect to take into account the preference of microhabitats by lichens and bryophytes (Hespanhol et al. 2011; Sulavik et al. 2021). Model residuals were inspected using the ***DHARMa*** package (Hartig 2022). As the ‘site’ had only two levels, it was treated as a fixed effect, since the low number of levels may lead to unreliable estimates of random variance components (Harrison et al. 2018).

## Results

A total of 45 bryophyte species and 7 lichen species were recorded in total. In the sampled plots, we recorded a maximum of 13 bryophytes species and a maximum of 6 lichen species, and a minimum of 0 species for both taxa. Bryophyte FD ranged from 0 to 0.60 (mean values of 0.33 for Pania della Croce and 0.10 for Monte Prado), while lichen FD ranged from 0 to 0.67 (mean values of 0.02 for Pania della Croce and 0.11 for Monte Prado).

**Fig. 3:**
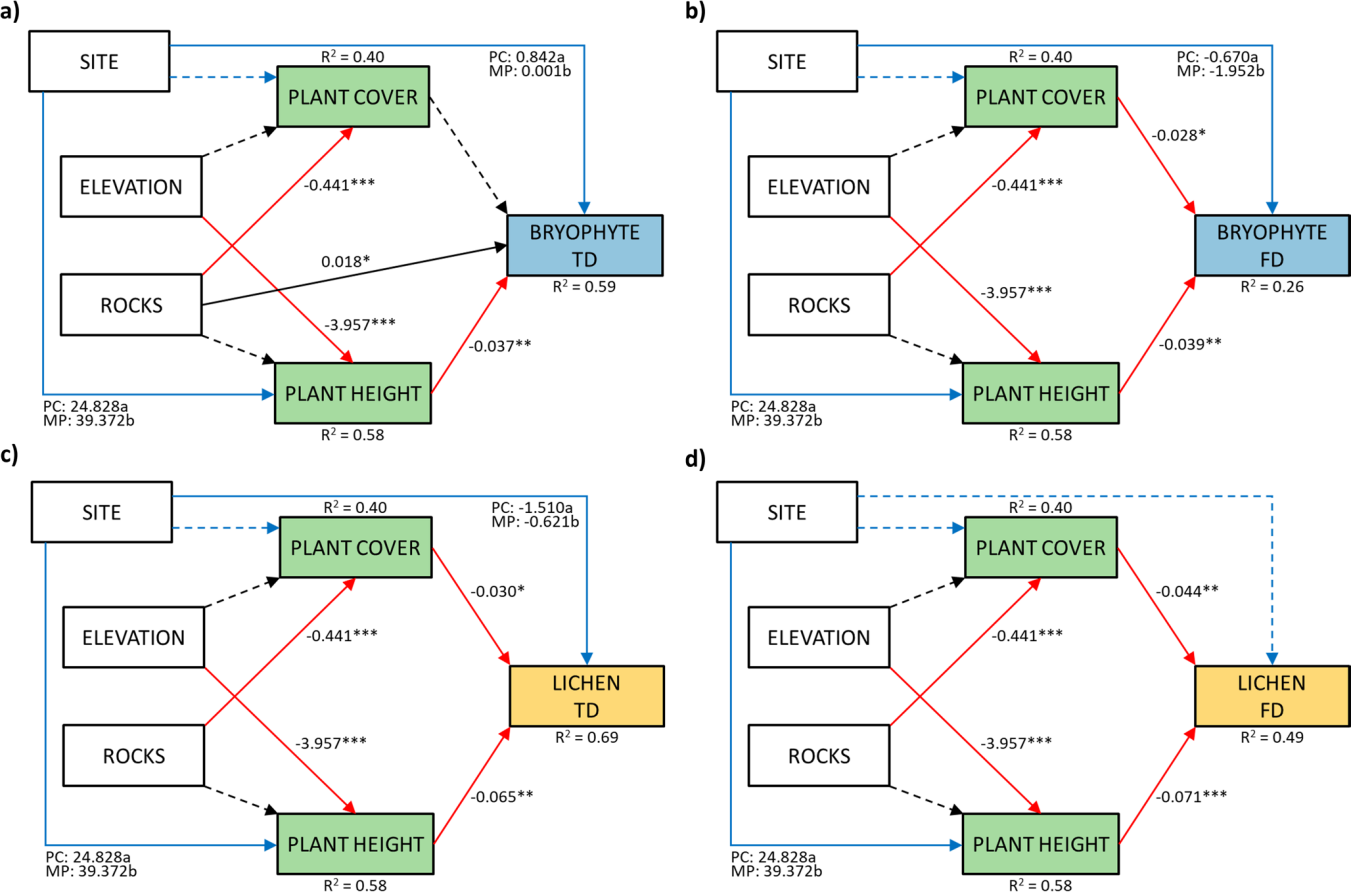
Structural equation models (piecewise SEMs) exploring the effects of environmental parameters and plant community characteristics on bryophyte (a and b) and lichen (c and d) diversity, for both taxonomic (a and c) and functional (b and d) facets. Solid arrows represent unidirectional significant relationships among variables (*p* < 0.05), black arrows denote positive relationships and red ones show negative relationships. Blue arrows indicate relationships between the site (categorical variable) and other variables. Dashed arrows show not significant relationships (*p* > 0.05). Standardized parameter estimates are given next to the arrows, where * *p* < 0.05, ** *p* < 0.01, and *** *p* < 0.001. Marginal R^2^ (based on fixed effects only) for component models are given above or below the box of respective response variable. The statistics of the models are: (a) Fisher’s C = 8.124, *p* = 0.087; (b) Fisher’s C = 8.523, *p* = 0.202; (c) Fisher’s C = 11.708, *p* = 0.069; (d) Fisher’s C = 6.217, *p* = 0.399

The piecewise structural equations models performed were statistically significant for both lichens and bryophytes communities and for both facets of diversity (Fig. 3). Moreover, SEMs explained 40% of the variation in vascular plant cover, due to the negative effect of rock abundance, and 58% of the variation in vascular plant height, attributable to the negative effect of elevation and a significant difference between sites.

Piecewise SEM explained 59% of the variation in bryophyte taxonomic diversity, which was attributable to positive effects of rocks and negative effects of plant height (Fig. 3a). By contrast, piecewise SEM explained the 26% of variation in bryophyte functional diversity and significantly affected by vegetation cover and height (Fig. 3b). Regarding lichens, SEM explained 69% of the variation in taxonomic diversity, attributable to negative effects of vascular plant coverage and height, and 49% of the variation in functional diversity (Fig. 3c), also attributable to negative effects of vascular plant coverage and height (Fig. 3d). The effect of different sites (and hence substrates) resulted significant for both facets of bryophyte diversity (Fig. 3a and b) and lichen taxonomic diversity (Fig. 3c).

## Discussion

The results obtained allowed us to disentangle the complex relationships between abiotic and biotic processes acting on the lichens and bryophytes components of the grassland communities investigated. Vegetation cover and height were the most important direct factor determining both facets of lichens and bryophytes diversity. In contrast, elevation had only an indirect effect, reducing vascular plant cover along the gradient and thus reducing competition.

The direct relationship between rock abundance and vascular plant cover confirms the negative effect of shallow soils and outcrops on vascular plant biomass production (e.g., Auestad et al. 2008; Bernard-Verdier et al. 2012; Yang et al. 2017; Mugnai et al. 2023). Moreover, a negative direct relationship between elevation and vegetation height was also found, suggesting that the environmental pressure, such as lower temperatures, may select for shorter vascular plant species (Molina-Venegas et al. 2016; Mota et al. 2018).

Regarding lichens and bryophytes diversity, we found that bryophyte taxonomic diversity was the only facet that is directly affected by abiotic parameters. In fact, the presence of rock outcrops (harsh patches for colonization of vascular plants) represents spaces with low competition from vascular plants (i.e., available niches which enhance a higher number of bryophyte species; see Bergauer et al. 2022), that create points of discontinuity in the homogeneous herb layer, creating microhabitats which promote richness and more variation in the composition of bryophyte communities (Hespanhol et al. 2011; Peñaloza-Bojacá et al. 2018; Ren et al. 2021). The negative effect of vascular plant competition was confirmed by the significant effect operated by vegetation height and cover, which was found to be a stronger factor in shaping bryophyte and lichen taxonomic diversity. In fact, being taller, vascular vegetation creates a shading effect on lichens and bryophytes, thus outcompeting them for light interception, and reducing their richness (Jägerbrand et al. 2012; Leppik et al. 2013; Fergus et al. 2017; Boch et al. 2018; Bergauer et al. 2022; Hagenberg et al. 2022). Hence, our results suggest that the elevational patterns showing an increase of grassland lichens and bryophytes diversity with elevation found by many authors (e.g., Baumann et al. 2016; Fontana et al. 2020) might be due to the progressive decrease of vascular plant biomass caused by environmental stresses like lower temperatures and shallower soils linked to elevation. Thus, our results suggest that competition is likely the main driver that directly assembles lichens and bryophytes taxonomic diversity, with the abiotic parameters (e.g., elevation and temperature) having only indirect effects through the shaping of vascular vegetation (Boch et al. 2018).

Similarly, the more pronounced effect of competition by vascular plants compared to environmental filtering operating along the elevational gradients was confirmed by the functional facets of lichens and bryophytes diversity. In fact, for both bryophytes and lichens, the vascular vegetation characteristics (vascular plant cover and height) were the only environmental parameters that shaped their functional diversity. These results suggest that, similar to taxonomic diversity, the patterns of functional diversity along the elevational gradients found by other authors for bryophytes (Henriques et al. 2017; Ah-Peng et al. 2014) and lichens (Bässler et al. 2016), might not be directly due to the environmental filtering of abiotic parameters but are subordinated by the competition of vascular plants. This is in accordance with previous studies, which found that lichens and bryophytes’ functional diversity is inversely related to the abundance of vascular plants, with the latter being the primary driver of biodiversity and having a significantly stronger influence than elevation, which only has indirect effects (Asplund et al. 2022). Nonetheless, it must be noted that as the trait values used here were collated from literature and not obtained via direct measurements of our sampled specimens. Hence, intraspecific trait variability along the gradient was not considered but future studies could explore the role of trait plasticity in lichens and bryophytes as a strategy to cope with competition.

Finally, all facets of biodiversity, except for lichen functional diversity, were significantly influenced by the site. This result might be related to the difference in bedrock type, as Monte Prado is characterized by siliceous bedrock, while Pania della Croce is calcareous. Different bedrock types are related to differences in the physical properties of the soil, such as percolation and water retention, and it is known to influence both plants (Nemer et al. 2021) and lichen diversity (Vallese et al. 2024). Such effects trigger positive feedback related to vegetation structures, which may affect habitat suitability for bryophytes and lichens. For example, in our study, calcareous substrates are characterized by the strong dominance of grass species (e.g., *Brachypodium* sp.), which forms tussocks that strongly outcompete lichens and bryophytes. Siliceous bedrock, on the other hand, presents a weaker dominance of such species, leaving more free microhabitats for lichens and bryophytes. Moreover, in our study, it has been possible to evaluate the role of factors and competition by vascular plants on lichens and bryophytes diversity without the effect of disturbance by grazing and trampling, which have been demonstrated to be a key process in the assemblage of bryophytes and lichens in grassland communities (Rai et al. 2012; Tripp et al. 2019).

## Conclusions

We demonstrated that lichens and bryophytes diversity in grassland habitats is directly shaped by the competition of vascular vegetation. In particular, the increasing taxonomic and functional diversity of lichen and bryophytes along the elevational gradient is due to the decrease in height and cover of vascular plants. Thus, abiotic factors such as elevation and availability of suitable microhabitats (i.e., abundance of rocks) affected lichens and bryophytes diversity only indirectly, mediated by their direct effects on vascular vegetation. This case study provides an important framework for understanding the lichens and bryophytes diversity along elevational gradients in grasslands. If further confirmed, our results may substantially advance our knowledge on the assembly rules of lichens and bryophytes communities, and the primary role of biotic factors in respect to abiotic ones. Nevertheless, further studies are needed to widen the findings at larger scales and depict general patterns underpinning the biodiversity of such organisms in grassland ecosystems.

## Supplementary Information

Below is the link to the electronic supplementary material.

Supplementary Material 1 (XLSX. 16.8 KB)

## Data Availability

The data used in this paper is accessible in the supplementary material.

## References

[CR1] Ah-Peng C, Flores O, Wilding N, Bardat J, Marline L, Hedderson TA, Strasberg D (2014) Functional diversity of subalpine bryophyte communities in an oceanic Island (La Réunion). Arct Antarct Alp Res 46(4):841–851

[CR2] Asplund J, Wardle DA (2017) How lichens impact on terrestrial community and ecosystem properties: how lichens impact on communities and ecosystems. Biol Rev 92:1720–1738. 10.1111/brv.12305 27730713 10.1111/brv.12305

[CR3] Asplund J, van Zuijlen K, Roos RE, Birkemoe T, Klanderud K, Lang SI, Wardle DA (2022) Divergent responses of functional diversity to an elevational gradient for vascular plants, bryophytes and lichens. J Veg Sci 33:e13105. 10.1111/jvs.13105

[CR4] Auestad I, Rydgren K, Økland RH (2008) Scale-dependence of vegetation-environment relationships in semi-natural grasslands. J Veg Sci 19:139–148. 10.3170/2007-8-18344

[CR5] Austrheim G, Hassel K, Mysterud A (2005) The role of life history traits for bryophyte community patterns in two contrasting alpine regions. Bryologist 108:259–271. 10.1639/0007-2745(2005)108[0259:TROLHT]2.0.CO;2

[CR6] Bässler C, Cadotte MW, Beudert B, Heibl C, Blaschke M, Bradtka JH, Langbehn T, Werth S, Müller J (2016) Contrasting patterns of lichen functional diversity and species richness across an elevation gradient. Ecography 39:689–698. 10.1111/ecog.01789

[CR7] Baumann E, Weiser F, Chiarucci A, Jentsch A, Dengler J (2016) Diversity and functional composition of alpine grasslands along an elevational transect in the Gran Paradiso National park (NW Italy). Tuexenia 36:337–358

[CR8] Bergauer M, Dembicz I, Boch S, Willner W, Babbi M, Blank-Pachlatko J, Catalano Chiara, Cykowska-Marzencka Beata, Gehler Jamyra, Guarino Riccardo, Keller Sabrina, Moysiyenko Ivan, Vynokurov Denys, Widmer Stefan, Dengler J (2022) Scale-dependent patterns and drivers of vascular plant, bryophyte and lichen diversity in dry grasslands of the Swiss inneralpine valleys. Alpine Botany 132(2):195–209. 10.1007/s00035-022-00285-y

[CR9] Bernard-Verdier M, Navas M-L, Vellend M, Violle C, Fayolle A, Garnier E (2012) Community assembly along a soil depth gradient: contrasting patterns of plant trait convergence and divergence in a mediterranean rangeland. J Ecol 100:1422–1433. 10.1111/1365-2745.12003

[CR10] Biurrun I, Pielech R, Dembicz I, Gillet François, Kozub Łukasz, Marcenò Corrado, Reitalu Triin, Van Meerbeek Koenraad, Guarino Riccardo, Chytrý Milan, Pakeman Robin J., Preislerová Zdenka, Axmanová Irena, Burrascano Sabina, Bartha Sándor, Boch Steffen, Bruun Hans Henrik, Conradi Timo, De Frenne Pieter, Essl Franz, Filibeck Goffredo, Hájek Michal, Jiménez‐Alfaro Borja, Kuzemko Anna, Molnár Zsolt, Pärtel Meelis, Pätsch Ricarda, Prentice Honor C., Roleček Jan, Sutcliffe Laura M. E., Terzi Massimo, Winkler Manuela, Wu Jianshuang, Aćić Svetlana, Acosta Alicia T. R., Afif Elias, Akasaka Munemitsu, Alatalo Juha M., Aleffi Michele, Aleksanyan Alla, Ali Arshad, Apostolova Iva, Ashouri Parvaneh, Bátori Zoltán, Baumann Esther, Becker Thomas, Belonovskaya Elena, Benito Alonso José Luis, Berastegi Asun, Bergamini Ariel, Bhatta Kuber Prasad, Bonini Ilaria, Büchler Marc‐Olivier, Budzhak Vasyl, Bueno Álvaro, Buldrini Fabrizio, Campos Juan Antonio, Cancellieri Laura, Carboni Marta, Ceulemans Tobias, Chiarucci Alessandro, Chocarro Cristina, Conti Luisa, Csergő Anna Mária, Cykowska‐Marzencka Beata, Czarniecka‐Wiera Marta, Czarnocka‐Cieciura Marta, Czortek Patryk, Danihelka Jiří, de Bello Francesco, Deák Balázs, Demeter László, Deng Lei, Diekmann Martin, Dolezal Jiri, Dolnik Christian, Dřevojan Pavel, Dupré Cecilia, Ecker Klaus, Ejtehadi Hamid, Erschbamer Brigitta, Etayo Javier, Etzold Jonathan, Farkas Tünde, Farzam Mohammad, Fayvush George, Fernández Calzado María Rosa, Finckh Manfred, Fjellstad Wendy, Fotiadis Georgios, García‐Magro Daniel, García‐Mijangos Itziar, Gavilán Rosario G., Germany Markus, Ghafari Sahar, del Giusso Galdo Gian Pietro, Grytnes John‐Arvid, Güler Behlül, Gutiérrez‐Girón Alba, Helm Aveliina, Herrera Mercedes, Hüllbusch Elisabeth M., Ingerpuu Nele, Jägerbrand Annika K., Jandt Ute, Janišová Monika, Jeanneret Philippe, Jeltsch Florian, Jensen Kai, Jentsch Anke, Kącki Zygmunt, Kakinuma Kaoru, Kapfer Jutta, Kargar Mansoureh, Kelemen András, Kiehl Kathrin, Kirschner Philipp, Koyama Asuka, Langer Nancy, Lazzaro Lorenzo, Lepš Jan, Li Ching‐Feng, Li Frank Yonghong, Liendo Diego, Lindborg Regina, Löbel Swantje, Lomba Angela, Lososová Zdeňka, Lustyk Pavel, Luzuriaga Arantzazu L., Ma Wenhong, Maccherini Simona, Magnes Martin, Malicki Marek, Manthey Michael, Mardari Constantin, May Felix, Mayrhofer Helmut, Meier Eliane Seraina, Memariani Farshid, Merunková Kristina, Michelsen Ottar, Molero Mesa Joaquín, Moradi Halime, Moysiyenko Ivan, Mugnai Michele, Naqinezhad Alireza, Natcheva Rayna, Ninot Josep M., Nobis Marcin, Noroozi Jalil, Nowak Arkadiusz, Onipchenko Vladimir, Palpurina Salza, Pauli Harald, Pedashenko Hristo, Pedersen Christian, Peet Robert K., Pérez‐Haase Aaron, Peters Jan, Pipenbaher Nataša, Pirini Chrisoula, Pladevall‐Izard Eulàlia, Plesková Zuzana, Potenza Giovanna, Rahmanian Soroor, Rodríguez‐Rojo Maria Pilar, Ronkin Vladimir, Rosati Leonardo, Ruprecht Eszter, Rusina Solvita, Sabovljević Marko, Sanaei Anvar, Sánchez Ana M., Santi Francesco, Savchenko Galina, Sebastià Maria Teresa, Shyriaieva Dariia, Silva Vasco, Škornik Sonja, Šmerdová Eva, Sonkoly Judit, Sperandii Marta Gaia, Staniaszek‐Kik Monika, Stevens Carly, Stifter Simon, Suchrow Sigrid, Swacha Grzegorz, Świerszcz Sebastian, Talebi Amir, Teleki Balázs, Tichý Lubomír, Tölgyesi Csaba, Torca Marta, Török Péter, Tsarevskaya Nadezda, Tsiripidis Ioannis, Turisová Ingrid, Ushimaru Atushi, Valkó Orsolya, Van Mechelen Carmen, Vanneste Thomas, Vasheniak Iuliia, Vassilev Kiril, Viciani Daniele, Villar Luis, Virtanen Risto, Vitasović‐Kosić Ivana, Vojtkó András, Vynokurov Denys, Waldén Emelie, Wang Yun, Weiser Frank, Wen Lu, Wesche Karsten, White Hannah, Widmer Stefan, Wolfrum Sebastian, Wróbel Anna, Yuan Zuoqiang, Zelený David, Zhao Liqing, Dengler J (2021) Benchmarking plant diversity of Palaearctic grasslands and other open habitats. J Veg Sci 32:e13050. 10.1111/jvs.13050

[CR11] Boch S, Prati D, Schöning I, Fischer M (2016) Lichen species richness is highest in non-intensively used grasslands promoting suitable microhabitats and low vascular plant competition. Biodivers Conserv 25:225–238. 10.1007/s10531-015-1037-y

[CR12] Boch S, Allan E, Humbert J-Y, Kurtogullari Y, Lessard-Therrien M, Müller J, Prati D, Rieder NS, Arlettaz R, Fischer M (2018) Direct and indirect effects of land use on bryophytes in grasslands. Sci Total Environ 644:60–67. 10.1016/j.scitotenv.2018.06.323 29980086 10.1016/j.scitotenv.2018.06.323

[CR13] Brooks ME, Kristensen K, van Benthem KJ, Magnusson A, Berg CW, Nielsen A, Skaug HJ, Maechler M, Bolker BM (2017) glmmTMB balances speed and flexibility among packages for zero-inflated generalized linear mixed modeling. R J 9(2):378–400. 10.32614/RJ-2017-066

[CR14] Bruun HH, Moen J, Virtanen R, Grytnes J, Oksanen L, Angerbjörn A (2006) Effects of altitude and topography on species richness of vascular plants, bryophytes and lichens in alpine communities. J Veg Sci 17:37–46. 10.1111/j.1654-1103.2006.tb02421.x

[CR15] Chase JM, McGill BJ, Thompson PL, Antão LH, Bates AE, Blowes SA, Dornelas M, Gonzalez A, Magurran AE, Supp SR, Winter M, Bjorkman AD, Bruelheide H, Byrnes JEK, Cabral JS, Elahi R, Gomez C, Guzman HM, Isbell F, Myers-Smith IH, Jones HP, Hines J, Vellend M, Waldock C, O’Connor M (2019) Species richness change across spatial scales. Oikos 128:1079–1091. 10.1111/oik.05968

[CR16] Clarke KR, Warwick RM (1998) A taxonomic distinctness index and its statistical properties. J Appl Ecol 35(4):523–531

[CR17] Cole HA, Newmaster SG, Bell FW, Pitt D, Stinson A (2008) Influence of microhabitat on bryophyte diversity in Ontario mixedwood boreal forest. Can J Res 38:1867–1876. 10.1139/X08-036

[CR18] Cornelissen JHC, Callaghan TV, Alatalo JM, Michelsen A, Graglia E, Hartley AE, Aerts R (2001) Global change and Arctic ecosystems: is lichen decline a function of increases in vascular plant biomass? J Ecol 89(6):984–994

[CR19] de Bello F, Lavorel S, Lavergne S, Albert CH, Boulangeat I, Mazel F, Thuiller W (2013) Hierarchical effects of environmental filters on the functional structure of plant communities: a case study in the French Alps. Ecography 36:393–402. 10.1111/j.1600-0587.2012.07438.x

[CR20] de Bello F, Carmona CP, Lepš J, Szava-Kovats R, Pärtel M (2016) Functional diversity through the mean trait dissimilarity: resolving shortcomings with existing paradigms and algorithms. Oecologia 180:933–940 26796409 10.1007/s00442-016-3546-0

[CR21] Deane-Coe KK, Stanton D (2017) Functional ecology of cryptogams: scaling from bryophyte, lichen, and soil crust traits to ecosystem processes. New Phytol 213:993–995. 10.1111/nph.14408 28079939 10.1111/nph.14408

[CR22] Dengler J, Janišová M, Török P, Wellstein C (2014) Biodiversity of palaearctic grasslands: a synthesis. Agric Ecosyst Environ 182:1–14. 10.1016/j.agee.2013.12.015

[CR23] Dengler J, Boch S, Filibeck G, Chiarucci A, Dembicz I, Guarino R, Henneberg B, Janišová M, Marcenò C, Naqinezhad A (2016) Assessing plant diversity and composition in grasslands across Spatial scales: the standardised EDGG sampling methodology. Bull Eurasian Dry Grassl Group 32:13–30

[CR24] Dettweiler-Robinson E, Bakker JD, Grace JB (2013) Controls of biological soil crust cover and composition shift with succession in sagebrush shrub-steppe. J Arid Environ 94:96–104

[CR25] Di Nuzzo L, Vallese C, Benesperi R, Giordani P, Chiarucci A, Di Cecco V, Di Martino L, Di Musciano M, Gheza G, Lelli C, Spitale D, Nascimbene J (2021) Contrasting multitaxon responses to climate change in mediterranean mountains. Sci Rep 11:4438. 10.1038/s41598-021-83866-x 33627718 10.1038/s41598-021-83866-x PMC7904820

[CR26] Ellis CJ, Yahr R, Hodkinson TR, Jones MB, Waldren S, Parnell JAN (2011) An interdisciplinary review of climate change trends and uncertainties: lichen biodiversity, arctic-alpine ecosystems and habitat loss. Climate change, ecology and systematics, 457–489

[CR27] Fergus AJ, Gerighausen U, Roscher C (2017) Vascular plant diversity structures bryophyte colonization in experimental grassland. J Veg Sci 28(5):903–914

[CR28] Fontana V, Guariento E, Hilpold A, Niedrist G, Steinwandter M, Spitale D, Nascimbene J, Tappeiner U, Seeber J (2020) Species richness and beta diversity patterns of multiple taxa along an elevational gradient in pastured grasslands in the European Alps. Sci Rep 10:12516. 10.1038/s41598-020-69569-9 32719437 10.1038/s41598-020-69569-9 PMC7385172

[CR29] Frenette-Dussault C, Shipley B, Hingrat Y (2013) Linking plant and insect traits to understand multitrophic community structure in arid steppes. Funct Ecol 27:786–792. 10.1111/1365-2435.12075

[CR30] Gagic V, Bartomeus I, Jonsson T, Taylor A, Winqvist C, Fischer C, Slade EM, Steffan-Dewenter I, Emmerson M, Potts SG, Tscharntke T, Weisser W, Bommarco R (2015) Functional identity and diversity of animals predict ecosystem functioning better than species-based indices. Proc R Soc B Biol Sci 282:20142620. 10.1098/rspb.2014.2620 10.1098/rspb.2014.2620 PMC4309003 25567651

[CR31] García de León D, Neuenkamp L, Gerz M, Oja E, Zobel M (2016) Secondary succession in alvar grasslands–do changes in vascular plant and cryptogam communities correspond? Folia Geobot 51:285–296

[CR32] Giordani P, Incerti G, Rizzi G, Rellini I, Nimis PL, Modenesi P (2014) Functional traits of cryptogams in mediterranean ecosystems are driven by water, light and substrate interactions. J Veg Sci 25:778–792. 10.1111/jvs.12119

[CR33] Glime JM (2017) Physiological ecology. In: Bryophyte ecology Volume 1. Ebook sponsored by Michigan Technological University and the International Association of Bryologists. http://digitalcommons.mtu.edu/bryophyte-ecology1/(25 March 2017)

[CR34] Gower JC (1971) A general coefficient of similarity and some of its properties. Biometrics 27:857–871

[CR35] Graglia E, Jonasson S, Michelsen A, Schmidt IK, Havström M, Gustavsson L (2001) Effects of environmental perturbations on abundance of subarctic plants after three, seven and ten years of treatments. Ecography 24(1):5–12

[CR36] Habel JC, Dengler J, Janišová M, Török P, Wellstein C, Wiezik M (2013) European grassland ecosystems: threatened hotspots of biodiversity. Biodivers Conserv 22:2131–2138. 10.1007/s10531-013-0537-x

[CR37] Hagenberg LWC, Vanneste T, Opedal ØH, Petlund HT, Björkman MP, Björk RG, Holien Håkon, Limpens Juul, Molau Ulf, Graae Bente Jessen, De Frenne P (2022) Vegetation change on mountaintops in northern Sweden: stable vascular-plant but reordering of lichen and bryophyte communities. Ecol Res 37(6):722–737

[CR38] Harrison XA, Donaldson L, Correa-Cano ME, Evans J, Fisher DN, Goodwin CE, Goodwin Cecily E.D., Robinson Beth S., Hodgson David J., Inger R (2018) A brief introduction to mixed effects modelling and multi-model inference in ecology. PeerJ 6:e4794. 10.7717/peerj.4794 29844961 10.7717/peerj.4794 PMC5970551

[CR39] Hartig F (2022) DHARMa: Residual diagnostics for hierarchical (multi-level/mixed) regression models. R Project. R package version 0.4.5

[CR40] Henriques DS, Rigal F, Borges PA, Ah-Peng C, Gabriel R (2017) Functional diversity and composition of bryophyte water-related traits in Azorean native vegetation. Plant Ecol Divers 10(2–3):127–137

[CR41] Hespanhol H, Séneca A, Figueira R, Sérgio C (2011) Microhabitat effects on bryophyte species richness and community distribution on exposed rock outcrops in Portugal. Plant Ecol Divers 4:251–264. 10.1080/17550874.2011.616546

[CR42] Hill MO, Preston CD, Bosanquet SDS, Roy DB (2007) BRYOATT: attributes of British and Irish mosses, liverworts and hornworts. Centre for Ecology and Hydrology

[CR43] Hurtado P, Prieto M, Aragón G, Escudero A, Martínez I (2019) Critical predictors of functional, phylogenetic and taxonomic diversity are geographically structured in lichen epiphytic communities. J Ecol 107:2303–2316. 10.1111/1365-2745.13189

[CR44] Ingerpuu N, Liira J, Pärtel M (2005) Vascular plants facilitated bryophytes in a grassland experiment. Plant Ecol 180:69–75. 10.1007/s11258-005-2508-0

[CR45] Jägerbrand AK, Kudo G, Alatalo JM, Molau U (2012) Effects of neighboring vascular plants on the abundance of bryophytes in different vegetation types. Polar Sci 6(2):200–208

[CR46] Karger DN, Conrad O, Böhner J, Kawohl T, Kreft H, Soria-Auza RW, Zimmermann NE, Linder HP, Kessler M (2017) Data from: Climatologies at high resolution for the earth’s land surface areas. Dryad Digital Repository. 10.5061/dryad.kd1d4 10.1038/sdata.2017.122 PMC5584396 28872642

[CR47] Klink van R, Boch S, Buri P, Rieder NS, Humbert JY, Arlettaz R (2017) No detrimental effects of delayed mowing or uncut grass refuges on plant and bryophyte community structure and phytomass production in low-intensity hay meadows. Basic Appl Ecol 20:1–9

[CR48] Lefcheck JS (2016) PiecewiseSEM: piecewise structural equation modelling in r for ecology, evolution, and systematics. Methods Ecol Evol 7:573–579. 10.1111/2041-210X.12512

[CR49] Leppik E, Jüriado I, Suija A, Liira J (2013) The conservation of ground layer lichen communities in Alvar grasslands and the relevance of substitution habitats. Biodivers Conserv 22:591–614

[CR50] Löbel S, Dengler J, Hobohm C (2006) Species richness of vascular plants, bryophytes and lichens in dry grasslands: the effects of environment, landscape structure and competition. Folia Geobot 41:377–393. 10.1007/BF02806555

[CR51] Martin P, Mallik AU (2017) The status of non-vascular plants in trait-based ecosystem function studies. Perspect Plant Ecol Evol Syst 27:1–8. 10.1016/j.ppees.2017.04.002

[CR52] Matos P, Geiser L, Hardman A, Glavich D, Pinho P, Nunes A, Soares AMVM, Branquinho C (2017) Tracking global change using lichen diversity: towards a global-scale ecological indicator. Methods Ecol Evol 8:788–798. 10.1111/2041-210X.12712

[CR53] Molina-Venegas R, Aparicio A, Lavergne S, Arroyo J (2016) How soil and elevation shape local plant biodiversity in a mediterranean hotspot. Biodivers Conserv 25:1133–1149. 10.1007/s10531-016-1113-y

[CR54] Mota GS, Luz GR, Mota NM, Silva Coutinho E, das Dores Magalhães Veloso M, Fernandes GW, Nunes YRF (2018) Changes in species composition, vegetation structure, and life forms along an altitudinal gradient of rupestrian grasslands in south-eastern Brazil. Flora 238:32–42. 10.1016/j.flora.2017.03.010

[CR55] Mugnai M, Wendt CF, Balzani P, Ferretti G, Cin MD, Masoni A, Frizzi F, Santini G, Viciani D, Foggi B, Lazzaro L (2021) Small-scale drivers on plant and ant diversity in a grassland habitat through a multifaceted approach. PeerJ 9:e12517. 10.7717/peerj.12517 35036118 10.7717/peerj.12517 PMC8711281

[CR56] Mugnai M, Trindade DP, Thierry M, Kaushik K, Hrček J, Götzenberger L (2022) Environment and space drive the community assembly of Atlantic European grasslands: insights from multiple facets. J Biogeogr 49(4):699–711

[CR57] Mugnai M, Ferretti G, Gesuelli E, Nuti L, Di Natale S, Corti E, Viciani D, Lazzaro L (2023) Site dependence of local variations in taxonomic and functional diversity of plant communities in semi-natural dry grasslands. Plant Ecol 224(1):95–111

[CR58] Müller J, Klaus VH, Kleinebecker T, Prati D, Hölzel N, Fischer M (2012) Impact of land-use intensity and productivity on bryophyte diversity in agricultural grasslands. PLoS One 7(12):e51520 23251563 10.1371/journal.pone.0051520 PMC3520803

[CR59] Nascimbene J, Marini L (2015) Epiphytic lichen diversity along elevational gradients: biological traits reveal a complex response to water and energy. J Biogeogr 42:1222–1232. 10.1111/jbi.12493

[CR60] Nemer D, Liancourt P, Delerue F, Randé H, Michalet R (2021) Species stress tolerance and community competitive effects drive differences in species composition between calcareous and siliceous plant communities. J Ecol 109(12):4132–4142

[CR61] Nimis PL, Martellos S (2020) ITALIC 6.0 - the information system on Italian lichens. http://dryades.units.it/italic. Accessed 20 Jan 2021

[CR62] Peñaloza-Bojacá GF, de Oliveira BA, Araújo CAT, Fantecelle LB, dos Santos ND, Maciel-Silva AS (2018) Bryophytes on Brazilian ironstone outcrops: diversity, environmental filtering, and conservation implications. Flora 238:162–174

[CR63] Pescador DS, de Bello F, Valladares F, Escudero A (2015) Plant trait variation along an altitudinal gradient in mediterranean high mountain grasslands: controlling the species turnover effect. PLoS One 10:e0118876. 10.1371/journal.pone.0118876 25774532 10.1371/journal.pone.0118876 PMC4361585

[CR64] Peters MK, Hemp A, Appelhans T, Behler C, Classen A, Detsch F, Ensslin A, Ferger SW, Frederiksen SB, Gebert F, Haas M, Helbig-Bonitz M, Hemp C, Kindeketa WJ, Mwangomo E, Ngereza C, Otte I, Röder J, Rutten G, Schellenberger Costa D, Tardanico J, Zancolli G, Deckert J, Eardley CD, Peters RS, Rödel M-O, Schleuning M, Ssymank A, Kakengi V, Zhang J, Böhning-Gaese K, Brandl R, Kalko EKV, Kleyer M, Nauss T, Tschapka M, Fischer M, Steffan-Dewenter I (2016) Predictors of elevational biodiversity gradients change from single taxa to the multi-taxa community level. Nat Commun 7:13736. 10.1038/ncomms13736 28004657 10.1038/ncomms13736 PMC5192166

[CR65] Rai H, Upreti DK, Gupta RK (2012) Diversity and distribution of terricolous lichens as indicator of habitat heterogeneity and grazing induced trampling in a temperate-alpine shrub and meadow. Biodivers Conserv 21:97–113

[CR66] Ren H, Wang F, Ye W, Zhang Q, Han T, Huang Y, Chu Guowei, Hui Dafeng, Guo Q (2021) Bryophyte diversity is related to vascular plant diversity and microhabitat under disturbance in karst caves. Ecol Indic 120:106947

[CR67] Roos RE, Zuijlen K, Birkemoe T, Klanderud K, Lang SI, Bokhorst S, Wardle DA, Asplund J (2019) Contrasting drivers of community-level trait variation for vascular plants, lichens and bryophytes across an elevational gradient. Funct Ecol 33:2430–2446. 10.1111/1365-2435.13454

[CR68] Seymour FA, Crittenden PD, Dyer PS (2005) Sex in the extremes: lichen-forming fungi. Mycologist 19:51–58. 10.1017/S0269915X05002016

[CR69] Sulavik J, Auestad I, Halvorsen R, Rydgren K (2021) Assessing recovery of alpine spoil heaps by vascular plant, bryophyte, and lichen functional traits. Restor Ecol 29:e13257. 10.1111/rec.13257

[CR70] Sundqvist MK, Sanders NJ, Wardle DA (2013) Community and ecosystem responses to elevational gradients: processes, mechanisms, and insights for global change. Annu Rev Ecol Evol Syst 44:261–280. 10.1146/annurev-ecolsys-110512-135750

[CR71] Tripp EA, Lendemer JC, McCain CM (2019) Habitat quality and disturbance drive lichen species richness in a temperate biodiversity hotspot. Oecologia 190:445–457 31093760 10.1007/s00442-019-04413-0

[CR72] Turtureanu PD, Palpurina S, Becker T, Dolnik C, Ruprecht E, Sutcliffe LME, Szabó A, Dengler J (2014) Scale- and taxon-dependent biodiversity patterns of dry grassland vegetation in Transylvania. Agric Ecosyst Environ 182:15–24. 10.1016/j.agee.2013.10.028

[CR73] Vallese C, Di Nuzzo L, Francesconi L, Giordani P, Spitale D, Benesperi R, Gheza Gabriele, Mair Petra, Nascimbene J (2024) Bedrock-dependent effects of climate change on terricolous lichens along elevational gradients in the Alps. J Fungi 10(12):836 10.3390/jof10120836 PMC11678017 39728332

[CR74] Violle C, Reich PB, Pacala SW, Enquist BJ, Kattge J (2014) The emergence and promise of functional biogeography. Proc Natl Acad Sci U S A 111:13690–13696. 10.1073/pnas.1415442111 25225414 10.1073/pnas.1415442111 PMC4183284

[CR75] Wietrzyk-Pełka P, Rola K, Patchett A, Szymański W, Węgrzyn MH, Björk RG (2021) Patterns and drivers of cryptogam and vascular plant diversity in glacier forelands. Sci Total Environ 770:144793 33497901 10.1016/j.scitotenv.2020.144793

[CR76] Wilson JB, Peet RK, Dengler J, Pärtel M (2012) Plant species richness: the world records. J Veg Sci 23:796–802. 10.1111/j.1654-1103.2012.01400.x

[CR77] Yang Y, Dou Y, An S (2017) Environmental driving factors affecting plant biomass in natural grassland in the loess Plateau, China. Ecol Indic 82:250–259. 10.1016/j.ecolind.2017.07.010

[CR78] Zulka KP, Abensperg-Traun M, Milasowszky N, Bieringer G, Gereben-Krenn B-A, Holzinger W, Hölzler G, Rabitsch W, Reischütz A, Querner P, Sauberer N, Schmitzberger I, Willner W, Wrbka T, Zechmeister H (2014) Species richness in dry grassland patches of Eastern Austria: a multi-taxon study on the role of local, landscape and habitat quality variables. Agric Ecosyst Environ 182:25–36. 10.1016/j.agee.2013.11.016

